# Outcomes and Risk Factors of Pyoderma Gangrenosum After Reduction Mammoplasty: A Systematic Review of the Literature and Case Report

**DOI:** 10.1007/s00266-025-04859-z

**Published:** 2025-05-19

**Authors:** Asmaa Ali Dahy, Rasha Mohamad Hassan, Ayman Altramsy, Amany Attalah Gad, Ali Mohamed Elameen, Ahmed Salem, Ahmed Abu-Elsoud

**Affiliations:** 1 Department of Plastic and Reconstructive Surgery, Faculty of Medicine for Girls, Al-Azhar University, Gameat Al Azhar, Nasr City, Cairo, Egypt; 2 Department of Dermatology and Venerology, Faculty of Medicine for Girls, Al-Azhar University, Cairo, Egypt; 3 Department of Plastic and Reconstructive Surgery, El-Sahel Teaching Hospital, Cairo, Egypt; 4 Department of Plastic and Reconstructive Surgery, Faculty of Medicine for Boys, Al-Azhar University, Cairo, Egypt; 5 Department of Plastic and Reconstructive Surgery, Faculty of Medicine (Assiut branch), Al-Azhar University, Cairo, Egypt

**Keywords:** Pyoderma gangrenosum, Breast, Reduction mammoplasty

## Abstract

**Background:**

Reduction mammoplasty is a common aesthetic and reconstructive breast procedure. Pyoderma gangrenosum (PG) is a rare inflammatory, non-infectious neutrophilic dermatosis. Post-surgical PG is characterized by ulcerative lesions at surgical sites commonly misdiagnosed as wound infection. The present systematic review was conducted to retrieve the potential factors and outcomes of post-reduction mammoplasty PG with a case report.

**Methods:**

An extensive systematic literature review was implemented from inception to 18 October 2024. All clinical studies that included patients with PG after reduction mammoplasty were included for systematic review.

**Results:**

A female patient presented ten days after reduction mammoplasty with bilateral wound dehiscence. Thirty-nine days after the operation, the wound showed a second dehiscence for which immunosuppressive drugs were prescribed. The patient responded to the latest regimen with complete healing of the ulcerative lesions of the right and left breasts. The present systematic review included 41 cases, encompassing the present case report. The median time to initial presentation of PG was 6.5 days. The median time to the diagnosis of PG was 15.5 days. The wound was healed by secondary intention among 26 (59.06%) patients. Skin grafting was performed for six (13.63%) patients, while three (6.81%) patients received skin substitutes.

**Conclusion:**

PG after reduction mammoplasty is a devastating condition associated with poor cosmetic outcomes. The condition is difficult to diagnose, and the majority of cases are misdiagnosed and potentially subjected to ineffective medical therapy and unnecessary surgical debridement that worsen the prognosis of PG. Patients with existing immunological disorders and patients with a history of breast cancer were at higher risk of developing PG after reduction mammoplasty. The risk of PG after reduction mammoplasty still existed despite undergoing previous breast or abdominal surgeries. Patients with post-reduction mammoplasty PG mostly presented with erythema, severe pain, and fever.

**Level of Evidence III:**

This journal requires that authors assign a level of evidence to each article. For a full description of these Evidence-Based Medicine ratings, please refer to the Table of Contents or the online Instructions to Authors www.springer.com/00266.

## Introduction

Reduction mammoplasty is a common aesthetic and reconstructive breast procedure. Approximately 100,000 patients in the USA are subject to reduced mammoplasty annually. Reduction mammoplasty aims to reduce breast volume and maintain nipple–areola complex (NAC) viability to recreate aesthetically pleasant breasts. The technique offers women greater comfort during activities by relieving weight-bearing pain, along with improving the women's self-esteem [1, 2]. However, reduction mammoplasty may be associated with considerable complications. This includes common complications such as infection, seroma, hematoma, wound breakdown, fat necrosis, and rare complications such as pyoderma gangrenosum (PG) [3].

PG is a rare inflammatory, non-infectious neutrophilic dermatosis. It is characterized by progressive painful necrotic lesions, including ulcers, pustules, or bullae. PG is commonly associated with systemic diseases, particularly rheumatoid arthritis, ulcerative colitis, and haematological malignancies [4]. The pathophysiology of PG is not entirely understood yet related to dysregulation of the adaptive and innate immune system and genetic predisposition [5]. PG are clinically presented as painful ulcers or lesions of non-specific histology, rendering the condition challenging to diagnose. Post-surgical PG is characterized by ulcerative lesions at surgical sites commonly misdiagnosed as wound infection. Post-surgical PG is extremely rare, with an estimated incidence of three to ten cases per million people. The disease is most commonly associated with breast surgeries, accounting for 25% of all post-surgical PG [6–9]. The rarity of the PG and the challenges with its diagnosis highlighted the ultimate need to identify the potential risk factors of post-surgical PG [10]. The delay in diagnosis and the inappropriate management of post-surgical PG may detrimentally impact the patient’s health status and quality of life [11].

The management of post-surgical PG needs timely diagnosis and effective management. Of note, post-surgical PG is commonly mismanaged with prolonged, ineffectual courses of antibiotics and surgical debridement of the wounds, which only worsens the condition. This may exacerbate the course of the disease by involving the surrounding unaffected skin, resulting in disfigurement of the aesthetically sensitive important areas like breasts [12, 13]. Accurate and timely diagnosis of post-surgical PG is imperative to the most effective therapies, which include immunosuppressants and immunomodulators. Whereas previously published reviews identified the risk factors and outcomes of post-surgical PG, the literature still needs to be more conclusive, particularly in the setting of reduction mammoplasty [7, 9, 14–18]. Therefore, the present systematic review was conducted to retrieve the potential risk factors and outcomes of post-reduction mammoplasty PG with reporting a case of post-surgical PG of the breasts. Such knowledge is crucial to identify patients at higher risk of post-reduction mammoplasty PG and implement the most efficient diagnostic and management workup. This could alleviate the negative consequences of post-surgical PG and avoid unnecessary harmful management for such patients.

## Materials and Methods

This systematic review was performed in accordance with PRISMA (Preferred Reporting Items for Systematic Reviews and Meta-Analysis) guidelines [19] and the recommendations of the Cochrane collaboration [20]. The study's methodology was documented in a protocol that was registered at the PROSPERO database (Registration number: CRD42024502073).

### Data Source

An extensive systematic literature review was implemented from inception to 18 October 2024, using the following databases: PubMed, Google Scholar, Web of Science (ISI), Scopus, SIGLE, Virtual Health Library (VHL), NYAM, Clinical trials, Controlled Trials (mRCT), EMBASE, Cochrane Collaboration, and WHO International Clinical Trials Registry Platform (ICTRP). The following keywords were used in every possible combination: ‘PG’, ‘Pyoderma gangrenosum’, ‘Breast’, ‘Mammary’, ‘Reduction’, ‘Mammoplasty.’ No restrictions were employed on patients’ age, sex, ethnicity, language, race, or place.

The search strategy implemented controlled vocabulary terms under the criteria of each searched database. A further manual search was executed to distinguish all additional conceivable articles that are not indexed. The cross-referencing method was carried out until no other relevant articles were detected.

### Study Selection

All clinical studies that included patients with PG after reduction mammoplasty were included for systematic review. Furthermore, studies in which data were unattainable to be extracted, review articles, guidelines, non-human studies, letters, comments, editorials, posters, and book chapters were excluded. Two reviewers performed the title, abstract, and full-text screening process to disclose the potentially relevant articles that met the eligibility criteria. The discussion dissolved the contradiction between the reviewers. The screening process and the causes of article exclusion were documented using PRISMA flowchart.

### Data Extraction

The following study characteristics data were extracted from the finally included articles: the title of the included study, the second name of the first author, year of publication, study design, study period, and study region. Baseline patients' demographic characteristics were extracted, including patients' age, ethnicity, race, history of PG, and comorbidities. The variables related to surgical procedure which included the pattern of reduction, side of surgery, operation time, post-operative wound dehiscence were extracted. The data related to PG were retrieved including wound-related data such as wound culture, ulceration, cellulitis, wound biopsies, and general manifestations. Histological findings and management-related data were extracted, including surgical debridement, immunosuppressants, and grafting. The outcomes of PG were retrieved including recurrence of PG, complications, and aesthetic outcomes.

### Descriptive Analysis

Categorical variables were expressed in the form of the number and percentage. Non-normally distributed data were reported using median and range. Normally distributed data were reported using mean and standard deviation (SD). Statistical analysis was performed using SPSS software version 26 for Windows (SPSS Inc., Chicago, IL, USA) [21]. Figures were renovated using GraphPad Prism (GraphPad Software, Inc., San Diego) software version 8 [22].

## Results

### Case Presentation

**Fig. 1:**
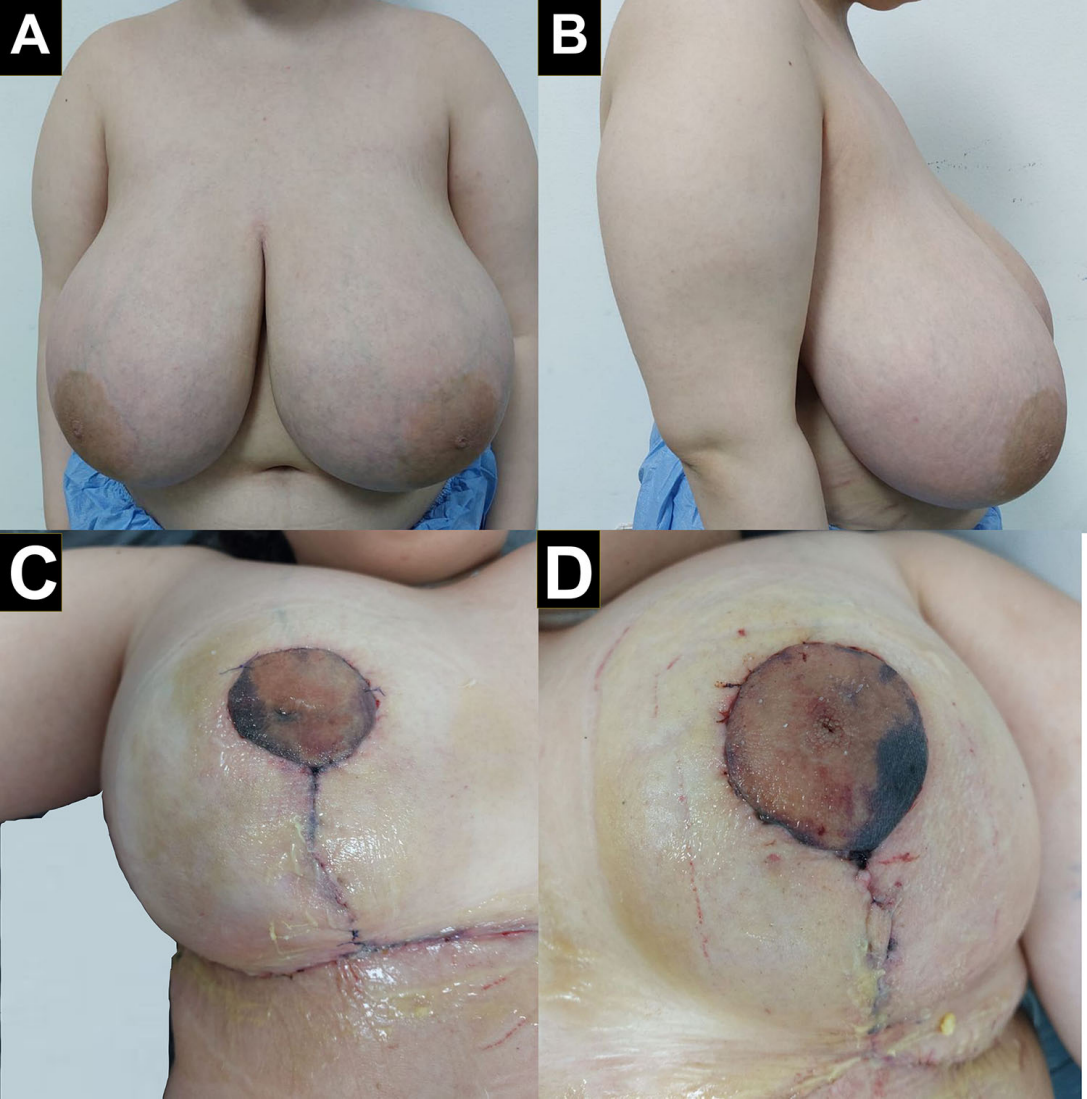
A female patient, 19 years old presented with bilateral breast hypertrophy. **A** Preoperative anterior view of both breasts. **B** Lateral view of the right breast preoperatively. **C** Immediate post-operative results of superior pedicle reduction mammoplasty of the left breast. **D** Immediate post-operative results of superior pedicle reduction mammoplasty of the left breast

**Fig. 2:**
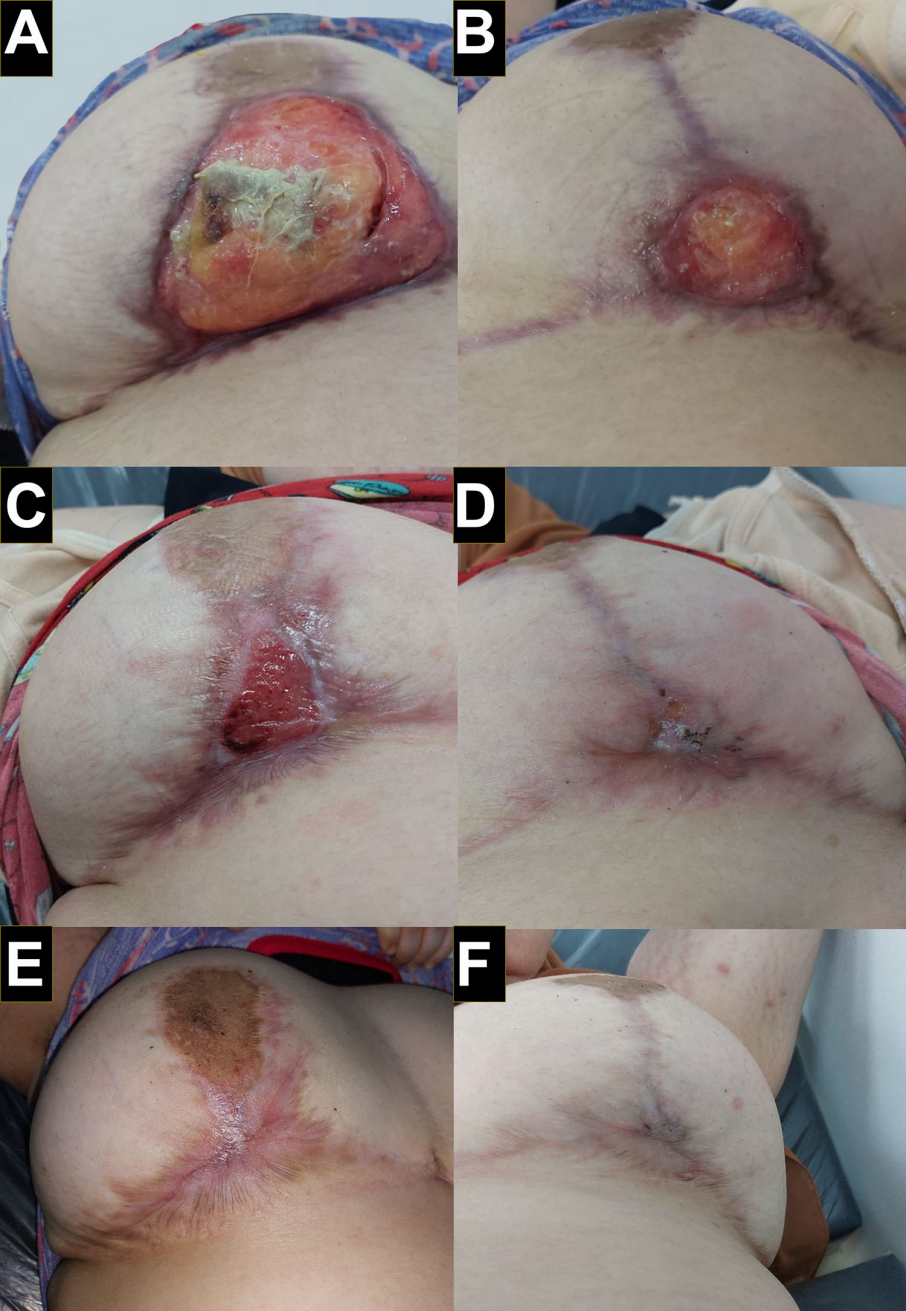
Thirty-nine days after the operation, the wound showed a second dehiscence. **A** Dehiscence at the right side measuring 8x10 cm sparing the nipple–areola complex. **B** Dehiscence at the right side measuring 5x4 cm sparing the nipple–areola complex. **C** Right breast after starting immunosuppressive therapy and immunomodulator showing epithelization and healing. **D** Right breast after starting immunosuppressive therapy and immunomodulator showing epithelization and healing. **E** Wound on the right side closed completely after approximately six months. **F** Wound on the left side closed completely after approximately five months

A female patient, 19 years old, non-diabetic, with no positive history of autoimmune diseases or family history of PG, presented with bilateral breast hypertrophy. The patient complained of mastalgia since breast hypertrophy. The visual analogue scale (VAS) for preoperative pain assessment was 5. The patient underwent bilateral reduction mammoplasty using the superior pedicle pattern. Preoperatively, the complete blood count (CBC) and the laboratory parameters were average, with no evidence of inflammation. Ten days after the operation, the patient developed a bilateral wound dehiscence, measuring 5×8 cm and 3×4 cm on the right and left sides, respectively. The dehiscence was associated with erythema, cellulitis, and superficial epidermolysis, sparing the nipples on both sides. The patient reported severe burning pain at the wound site and the surrounding tissues, with a score of 8–10 on the VAS for pain assessment. The punch biopsy showed predominant neutrophilic infiltration. Blood, urine, and wound cultures were negative. The wound was debrided and cleaned, local wound care was applied along with systemic antibiotics, and the wound was closed with primary sutures. Thirty-nine days after the operation, the wound showed a second dehiscence with a more progressive wound, measuring 8 × 10 cm and 5 × 4 cm on the right and left sides. The immunological markers showed a low positive antinuclear antibody (ANA) assay. Herein, the suspicion of PG was raised, and the medical treatment was started accordingly. At this time, the patient received corticosteroids (1 ml/kg) with a total of 70 ml per day and colchicine, which revealed poor response and recurrent PG after three months of therapy. The corticosteroid dose was adjusted, and the patient received an additional 100 mg of cyclosporin. The wound was treated locally with topical 0.1% tacrolimus, timolol, and collagenase agents for three months. The patients responded to the latest regimen in which the wound on the left side closed completely after five months. The wound on the right side closed completely after approximately six months. The patient experienced mild pain with VAS of 1–2 localized to the wound scar (Figs. 1, 2).

### Systematic Review of the Literature

**Fig. 3:**
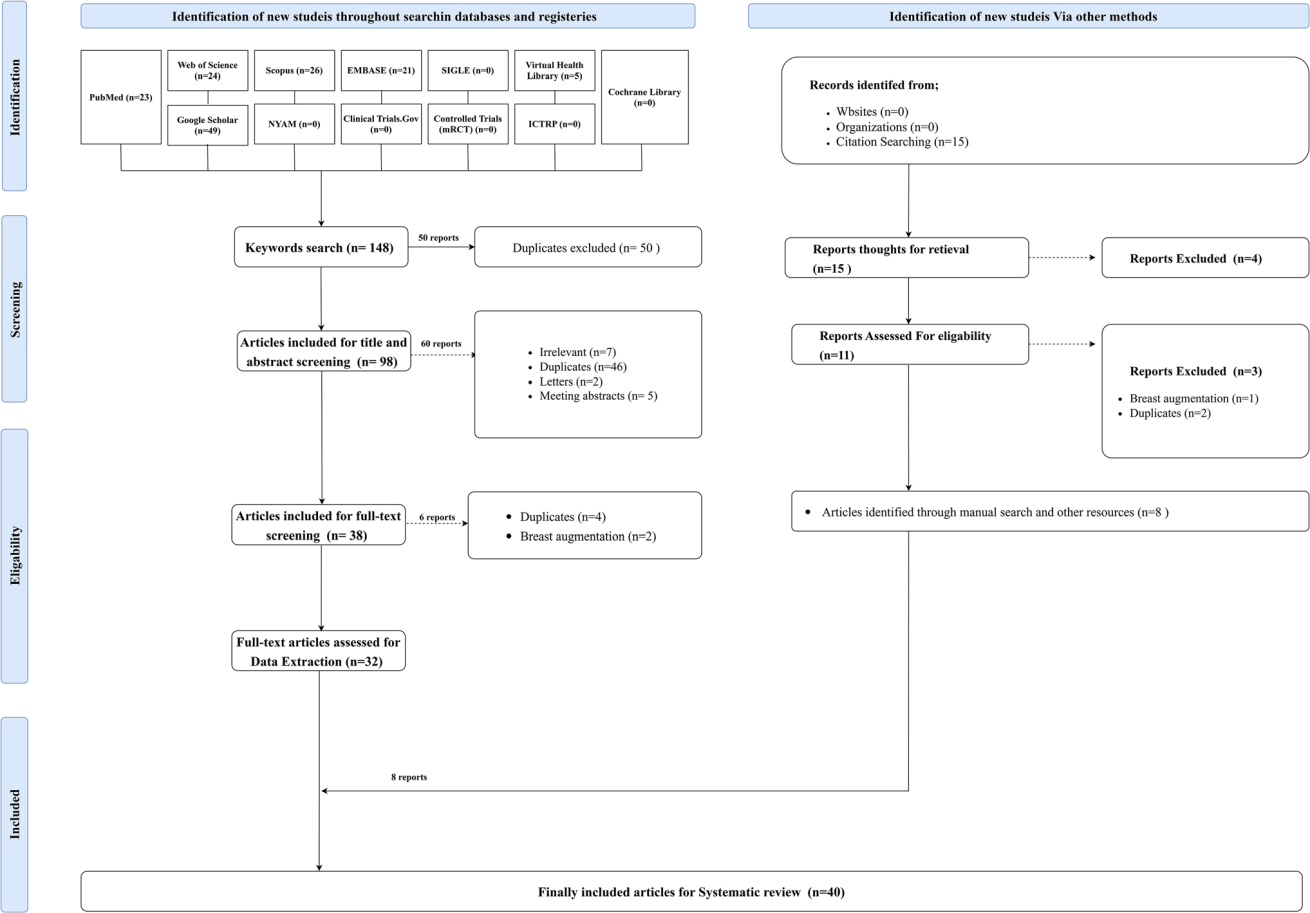
PRISMA 2020 flow diagram for updated systematic reviews which included searches of databases, registers, other sources, and screening.

A systematic review of the literature revealed 148 articles. Of them, 50 articles were duplicates, and 98 were included for title and abstract screening. Thirty-eight articles were eligible for full-text screening, and 32 articles were included for data extraction. Eight studies were identified through manual searching, resulting in 40 studies finally being included for systematic review and descriptive analysis in addition to the present case report (Fig. 3).

### Demographic Characteristics and Factors Associated with Pyoderma Gangrenosum

The present systematic review included 41 studies, encompassing the present case report [12, 23–59]. These studies included 44 cases with PG after reduction mammoplasty. Twelve studies included patients from the USA, six from Brazil, and five from the UK. The age of the included patients ranged from 17 to 61 years, with a mean age of 39.043 ± 13.85 years. Four (9.09%) patients had ulcerative colitis or Crohn’s disease, and three (6.81%) were obese. Three (6.81%) patients had a history of breast cancer, and two (4.54%) had hypothyroidism. Nine (20.45%) patients underwent previous operations without developing PG previously. Five of the patients underwent previous abdominal surgeries, and two patients were subjected to previous breast surgeries. Furthermore, 41 (93.81%) were subjected to bilateral reduction mammoplasty. The design of the pedicle was an inferior pattern in 13 (29.54%) patients, superomedial in three (6.81%) patients, and superior in three (6.81%) patients (Tables 1, 3).

**Table 1:** Demographic characteristics of the included studies

Study ID		Study region	Follow-up period	Age	Comorbidities	Previous operations	Pattern of reduction	Side of breast reduction	Days to initial presentation	Time to diagnosis	Initial presentation			Debridement
											Fever	Erythema	Severe Pain	
1	Gonçalves et al., 2002 [37]	France	NR	41	No	Thyroidectomy	NR	Bilateral	11	4 months	Yes	Yes	Yes	No
2	Goshtasby et al., 2010 [38]	USA	2 Years	49	Obesity	Total colectomy	Inferior pedicle technique	Bilateral	6	14 days	Yes	Yes	Yes	Yes
3	Bahadirli et al., 2019 [25]	Turkey	8 months	38	No	Rhinoplasty	NR	Bilateral	4	7 days	Yes	Yes	Yes	No
4	Gulyas et al., 2003 [41]	Australia	NR	57	Hypertensive	Appendectomy	NR	Bilateral	6	NR	Yes	Yes	Yes	Yes
5	Ozgenel et al., 2012 [62]	Turkey	Three months	38	No	No	Inferior pedicle technique	Bilateral	14	6 weeks	No	Yes	Yes	No
6	Tanini et al., 2017 [57]	Italy	NR	39	No	Gastric bypass	Inferior pedicle technique	Bilateral	4	11 days	Yes	Yes	Yes	Yes
7	Obinwanne et al., 2024 [46]	USA	10 months	55	No	No	NR	Bilateral	7	17 days	Yes	Yes	Yes	No
8	Yamaki et al., 2023 [59]	Brazil	NR	50	No	No	I Liacyr Ribeiro pedicle.	Bilateral	7	NR	No	Yes	Yes	No
9	Gaspar-Junior et al., 2018 [36]	Brazil	NR	56	No	No	NR	Bilateral	NR	3 Months	No	Yes	Yes	Yes
10	CALDERÓN et al., 2013 [28]	Salvador	one months	54	No	No	Inferior pedicle technique	Bilateral	NR	NR	No	Yes	Yes	Yes
			NR	23	No	No	Inferior pedicle technique	Bilateral	5	NR	No	Yes	Yes	Yes
			NR	21	No	No	Inferior pedicle technique	Bilateral	7	NR	No	Yes	Yes	Yes
11	Alvarez et al., 2020 [24]	Spain	NR	51	No	Gastric bypass and abdominoplasty	Superior pedicle	Bilateral	20	80 Days	NR	NR	NR	Yes
12	Peoples et al., 2021 [47]	USA	NR	25	PCOS, fibromyalgia, and POTS	No	NR	Bilateral	3	10 days	Yes	Yes	Yes	Yes
13	Saki et al., 2019 [63]	Germany	NR	17	Anorexia nervosa	No	Inverted T incision with superomedial pedicle breast reduction	Bilateral	7	17 days	No	Yes	Yes	Yes
14	Grillo et al., 2012 [39]	Brazil	NR	58	Hypertension, hepatitis B. A	No	Superomedial pedicle, inverted T technique	Bilateral	4	10 days	Yes	Yes	Yes	Yes
15	Širokáwt al., 2021 [55]	Czech Republic	6 months	39	No	No	NR	Bilateral	5	NR	Yes	Yes	Yes	Yes
16	Fong et al., 2021 [35]	Brazil	NR	46	Hepatic focal nodular hyperplasia	No	Superomedial pedicle, inverted T technique	Bilateral	5	7 weeks	Yes	Yes	Yes	Yes
17	Lee et al., 2024 [44]	USA	NR	61	No	No	NR	Bilateral	7	NR	Yes	Yes	Yes	Yes
18	Costa et al., 2013 [31]	Portugal	10 months	19	Anorexia nervosa	Inguinal hernia repair and bilateral otoplasty	NR	Bilateral	2	NR	NR	Yes	Yes	Yes
19	Lifchez et al., 2002 [45]	USA	3 months	38	Hypothyroidism, neurogenic bladder, and depression	No	NR	Bilateral	8	18 days	NR	Yes	Yes	Yes
20	Soares et al., 2001 [56]	Brazil	NR	31	No	NR	NR	Bilateral	3	8 days	NR	Yes	Yes	Yes
21	Clugston et al., 1991 [30]	Canada	NR	37	NR	NR	NR	NR	NR	NR	NR	NR	NR	NR
22	Segaran et al., 2013 [53]	UK	NR	31	No	No	NR	Bilateral	7 years	7 years	No	No	No	No
23	Horner et al., 2004 [43]	UK	NR	21	No	No	Inferior pedicle	Bilateral	9	3 months	No	Yes	Yes	Yes
24	Gudi et al., 2000 [40]	UK	NR	34	Hypothyroidism	NR	NR	Bilateral	14	1.5 years	NR	NR	NR	Yes
25	Doren et al., 2014 [33]	USA	NR	61	Breast Cancer	Partial Mastectomy and Radiation	A Wise pattern incision t	Right	4	12 days	NR	Yes	Yes	Yes
26	Simon et al., 2006 [54]	USA	6 Years	35	Eczema and a previous diagnosis of ulcerative colitis	NR	Inferior pedicle reduction	Bilateral	4	NR	No	Yes	Yes	Yes
27	Brucato et al., 2024 [27]	Switzerland	36 Months	58	Breast Cancer	Conservative Breast surgery	NR	Left	3	9 days	NR	Yes	Yes	Yes
28	Grau Salvat et al., 1998 [51]	Spain	NR	27	No	No		Bilateral	4	10 days	Yes	Yes	Yes	Yes
29	Pontes et al., 2021 [48]	Brazil	2 months	17	No	No	Inferior pedicle reduction	Bilateral	7	7	Yes	Yes	Yes	No
30	Rand et al., 1988 [49]	USA	NR	19	No	No	NR	Bilateral	4	NR	NR	Yes	Yes	No
31	Schöfer et al., 2002 [52]	Germany	22 weeks	24	No	No	NR	Bilateral	5	NR	NR	NR	NR	NR
32	Berry et al., 2003 [26]	UK	6 weeks	54	No	No	NR	Bilateral	4	6 days	Yes	Yes	Yes	No
33	Davis et al., 2006 [32]	USA	1 year	58	Ulcerative colitis	NR	NR	Bilateral	21	NR	NR	NR	NR	Yes
34	Ouazzani et al., 2007 [12]	Belgium	5 months	48	No	No	NR	Bilateral	14	NR	NR	NR	NR	NR
35	Carrasco et al., 2012 [29]	Spain	Four months	51	Breast Cancer	NR	NR	Bilateral	2	2 days	NR	NR	NR	Yes
36	Abtahi-naeini et al., 2016 [23]	Iran	NR	21	No	No	NR	Bilateral	20	NR	NR	NR	NR	Yes
37	Touil et al., 2017 [58]	UK	One year	48	No	No	Inferior pedicle technique	Bilateral	6	NR	Yes	Yes	Yes	Yes
38	Hammond et al., 2020 [42]	USA	NR	35	Ulcerative colitis	No	superior pedicle	Bilateral	10	NR	NR	NR	NR	NR
39	Rich et al., 2021 [50]	USA	NR	44	Obesity and COVID-19	No	NR	Bilateral	NR	NR	NR	NR	NR	NR
40	Edinger et al., 2022 [34]	USA	NR	35	Crohn’s disease, smoking	NR	Inferior pedicle	Bilateral	10	Eleven months	No	Yes	Yes	Yes
				24	obesity	NR	Inferior pedicle	Bilateral	7	13 days	Yes	Yes	Yes	Yes
41	Present study	Egypt	6 months	19	No	No	Superior pedicle pattern	Bilateral	10	39 days	No	Yes	Yes	Yes

*PCOS* polycystic ovarian syndrome, *POTS* postural orthostatic tachycardia syndrome

### Clinical Presentation and Management of Pyoderma Gangrenosum

**Fig. 4:**
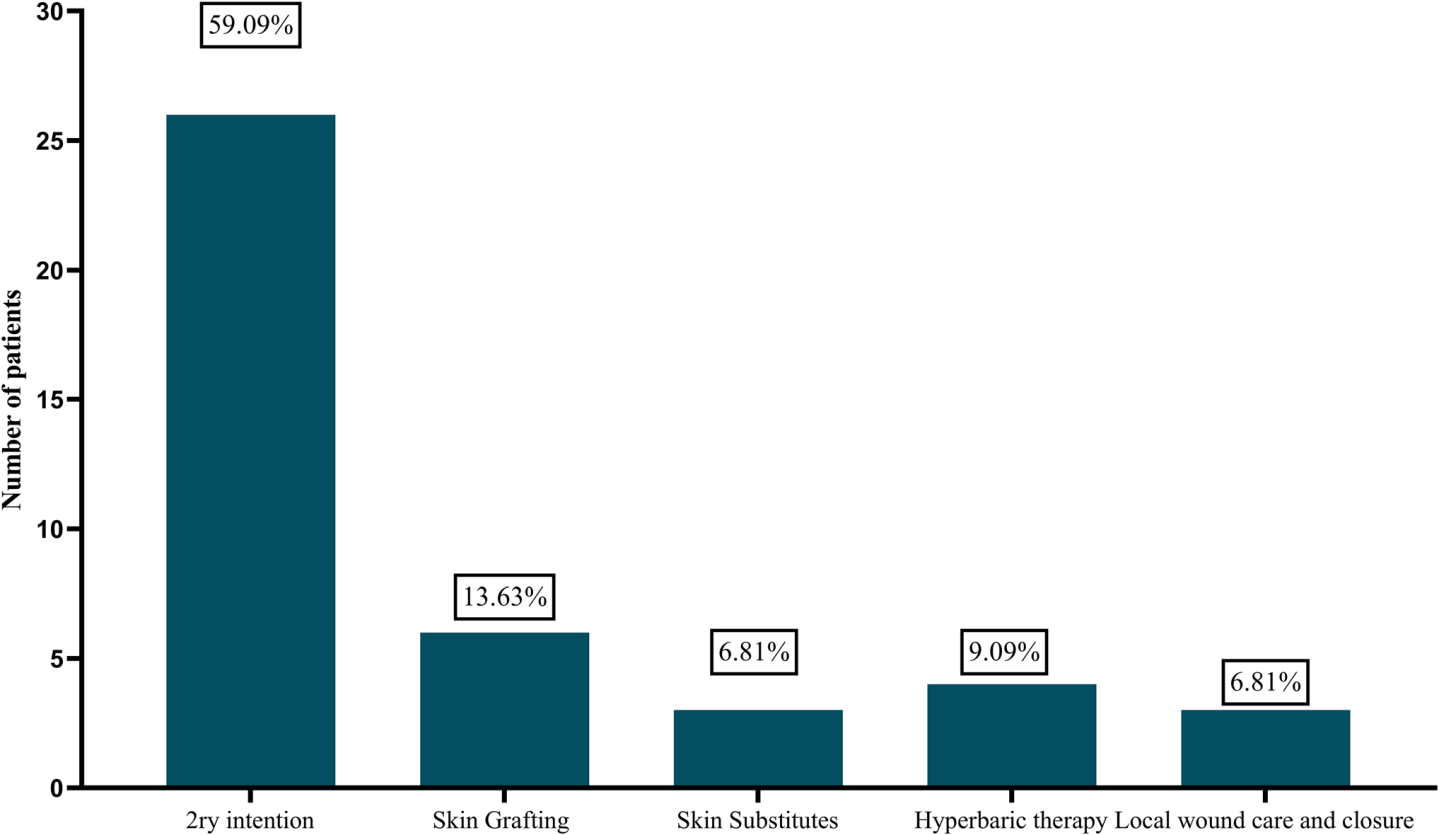
Bar chart showing the interventions for pyoderma gangrenosum after reduction mammoplas.

The median time to initial presentation of PG was 6.5 days (2 days, 7 years). The median time to the diagnosis of PG was 15.5 days (2 days, 7 years). The commonest initial presentation was erythema in 33 (75%) patients, severe pain in 33 (75%) patients, and fever in 16 (36.36%) patients. The initial management of the condition was based on local and systemic antibiotics. Thirty (68.18%) patients underwent surgical debridement before the diagnosis of pyoderma gangrenosum. The results of wound culture were negative in 25 (56.81%) specimens and were positive for Staphylococcus aureus in five (11.36%) specimens. Two (4.54%) specimens were positive for Corynebacterium striatum. The ANA was elevated among three (6.81%) patients, and hypogammaglobulinemia was detected among one (2.1%) patient (Tables 2, 3 and Fig. 4).

**Table 2:** Management and outcomes of pyoderma gangrenosum after reduction mammoplasty

Study ID		Initial treatment	Culture	Definitive medical treatment	Definitive surgical management	Recurrence	Outcomes
1	Gonçalves et al., 2002 [37]	Triple intravenous antibacterial drug with Tazocilline, Vancomycine and Gentamicine	Negative	Corticotherapy treatment with methylprednisolone in a dose of 1 milligram per kilogram and a day of 60 milligrams is recommended for 24 hours a day.	STSG	No	NR
2	Goshtasby et al., 2010 [38]	Intravenous and local antibiotherapy	Negative	Oral prednisone 60 mg daily (1 mg/kg/d).	Integra and Vac, and Skin Graft	No	Survival of graft and need for tissue expansion and mastopexy
3	Bahadirli et al., 2019 [25]	Intravenous and local antibiotherapy	Negative	80 mg/day oral prednisone	Synthetic porcine xenograft and then autograft	No	Distorted
4	Gulyas et al., 2003 [41]	Antibiotics and Hyperbaric oxygen therapy	Negative	Prednisolone, 50 mg daily)	Skin Grafting	No	Survived Skin Grafts
5	Ozgenel et al., 2012 [62]	Antibiotics	Negative	Oral cyclos porin A 250 mg daily and a mixture of silverdine, bepanthene and prednol cream topically.	secodnery intention	No	Completely healed
6	Tanini et al., 2017 [57]	Antibiotics	Negative	IV methylprednisolone (1.2 mg/kg/die)	VAC and primary wound closure	No	Wound closure
7	Obinwanne et al., 2024 [46]	Antibiotics	Corynebacterium striatum	High-dose prednisone, topical mupirocin, topical hydrocortisone	secodnery intention	No	Well-healed scars with decreased pain and improved quality of life
8	Yamaki et al., 2023 [59]	Antibiotics	Negative	Corticoid therapy with prednisone 100 mg/day and local skin care	Non-adherent cellulose acetate mesh	No	NR
9	Gaspar-Junior et al., 2018 [36]	Antibiotics	Negative	Corticosteroids 20 mg of prednisone for 1 week, then 15 mg for another week, then 10 mg for 1 week, and then 5 mg for 3 weeks, totaling 6 weeks of corticosteroid treatment	Hyperbaric therapy and secodnery intention	No	NR
10	CALDERÓN et al., 2013 [28]	Antibiotics No No	NR	Prednisone and dressings with dapsone	secodnery intention	No	NR
			Negative	Prednisone, azathioprine and dressings of the affected area with Bio-Piel (chitosan)	secodnery intention	No	Distorted
			Staphylococcus aureus	Prednisone, cotrimoxazole and Bio-Piel	secodnery intention	No	NR
11	Alvarez et al., 2020 [24]	Reoperation, Skin grafting, and VAC	NR	Oral corticosteroids	secodnery intention	No	Almost complete healing in breast wounds
12	Peoples et al., 2021 [47]	Antibiotics	Negative	Immunomodulatory medications: 10 mg prednisone (Deltasone, Pfizer) and 100 mg cyclosporine (Gengraf, AbbVie Inc.) daily.	Gender-affirming top surgery	No	NR
13	Saki et al., 2019 [63]	Antibiotics	Negative	Corticosteroid–tacrolimus therapy	NR	No	NR
14	Grillo et al., 2012 [39]	Antibiotics	NR	Prednisone 60 mg/day	hyperbaric oxygen therapy and local wound care	No	Distorted
15	Širokáwt al., 2021 [55]	Antibiotics	NR	Methylprednisolone was administered intravenously at a dose of 120 mg/day for 6 days, followed by prednisone (Prednison®, Zentiva, Czech Republic) at a dose of 50 mg/day.	secodnery intention	No	Flat hypertrophic scars remained in the lower quadrants of the breasts
16	Fong et al., 2021 [35]	Antibiotics	NR	oral prednisolone 60 mg per day	secodnery intention	No	bilateral breast wounds healed
17	Lee et al., 2024 [44]	NR	NR	Prednisone with planned taper and adalimumab for treatment	NR	No	Near complete reepithelization of both breasts
18	Costa et al., 2013 [31]	Antibiotics	Negative	Oral prednisolone 40 mg per day	NR	No	Scars and breast contour were acceptable
19	Lifchez et al., 2002 [45]	Antibiotics	Negative	vitamin A supplements to promote wound healing while on steroids	STSG	No	Grafts excellent take
20	Soares et al., 2001 [56]	Antibiotics	NR	10 mg of dexamethasone was administered between 6 and 8 days after the second surgery, prednisone (20 mg) and diaminodiphenyl sulfone (50 mg) for 15 days	Hyperbaric Oxygen and secondary Intention	No	Complete wound healing.
21	Clugston et al., 1991 [30]	NR	NR	Intralesional triamcinolone	NR	No	NR
22	Segaran et al., 2013 [53]	No	NR	Prednisolone 30 mg twice daily and oxytetracycline hydrochloride and hydrocortisone acetate ointment	NR	Yes Four years later	NR
23	Horner et al., 2004 [43]	Yes	Staphylococcus aureus, coliforms, Pseudomonas aeruginosa	Oral prednisolone 60 mg daily and topical betnovate cream twice daily	secodnery intention	No	NR
24	Gudi et al., 2000 [40]	Antibiotics	Negative	Topical clobetasol propionate 0.05%		No	Scarring around her nipples
25	Doren et al., 2014 [33]	Antibiotics	Negative	High-dose IV steroids, oral prednisone taper and topical tacrolimus	secodnery intention	No	Exhibited healed wounds and no signs of continued inflammation or infection
26	Simon et al., 2006 [54]	Antibiotics	Staphylococcus aureus with Mult resistant patterns	High-dose oral prednisone therapy	secodnery intention	No	NR
27	Brucato et al., 2024 [27]	Antibiotics	NR	Systemic corticosteroid therapy was immediately started on day 9 after surgery, administrating intravenous methylprednisolone for 4 days (125 mg/day), followed by oral prednisone (50 mg/day) that was gradually decreased over a period of 2 months.	secodnery intention	No	NR
28	Grau Salvat et al., 1998 [51]	Antibiotics	Negative	Oral cyclosporine (5 mg/kg/day) and methylprednisolone (0.5 mg/kg/day)	secodnery intention	No	Residual Scarring
29	Pontes et al., 2021 [48]	Antibiotics	NR	40mg/day prednisolone plus amoxicillin with clavulanate 875+125mg 12/12h	Daily Dressings and hyperbaric therapy	No	Good healing of the extensive lesions
30	Rand et al., 1988 [49]	Antibiotics	Negative	Methylprednisolone 1g IV followed by 40mg/day prednisolone	secodnery intention	No	NR
31	Schöfer et al., 2002 [52]	Antibiotics	Staphylococcus aureus and Escherichia coli	Cyclosporin treatment was initiated at 5 mg/ kg per day and gradually reduced to 2 mg/kg per day	secodnery intention	No	Complete healing
32	Berry et al., 2003 [26]	Antibiotics	NR	Ciclosporin 5 mg/kg	secodnery intention	No	Wounds had epithelialized
33	Davis et al., 2006 [32]	Antibiotics	Candida and Corynebacterium	Prednisone (60 mg/d; approximately 0.7 mg/kg/d)	secodnery intention	No	Wounds had epithelialized
34	Ouazzani et al., 2007 [12]	Antibiotics	Negative	Methylprednisolone 16 mg/day orally	secodnery intention	No	NR
35	Carrasco et al., 2012 [29]	Antibiotics	Negative	Immunosuppressive drugs (cyclosporin 100 mg 12 hourly orally	secodnery intention	No	Wound healed, leaving a large scar.
36	Abtahi-naeini et al., 2016 [23]	Antibiotics	Negative	50 mg/day oral prednisolone	secodnery intention	No	The wound healed with a fine atrophic scarring
37	Touil et al., 2017 [58]	Antibiotics	Negative	Oral prednisolone (40 mg once daily) and ranitidine (150 mg twice daily for gastroprotection)	secodnery intention	No	NR
38	Hammond et al., 2020 [42]	NR	Negative	Corticosteroids	NR	NR	NR
39	Rich et al., 2021 [50]	NR	NR	Methylprednisolone	secodnery intention	No	Distortion of both breasts
40	Edinger et al., 2022 [34]	Antibiotics	Pan-sensitive Staphylococcus aureus Negative	40 mg prednisone daily, decreasing by 10 mg every 5 days down to 5 mg daily	secodnery intention	No	NR
				Cyclosporine 175 mg twice daily (the upper end of 4–5 mg/kg/day dosing) a prednisone taper of 60 mg daily for 1 week followed by 40 mg daily for 1 week ending with 20 mg daily for 1 week.	secodnery intention	No	NR
41	Present study	Antibiotics	Negative	Corticosteroids (1ml/kg) with a total of 70 ml per day and colchicine	secodnery intention	No	Scarring around her nipples

*PCOS* polycystic ovarian syndrome, *POTS* postural orthostatic tachycardia syndrome, *STSG* split thickness skin graft, *VAC* vacuum-assisted closure

**Table 3:** Clinical characteristics of previously reported patients with pyoderma gangrenosum after reduction mammoplasty

Variables	Mean±SD/Median(Range)/Number (%)
*Age (Years)*	39.043 ±13.85
*Comorbidities*	
Ulcerative colitis or Crohn’s disease	4 (9.09%)
Obesity	3 (6.81%)
Hypertension	2 (4.54%)
Anorexia nervosa	2 (4.54%)
Hepatic diseases	2 (4.54%)
Hypothyroidism	2 (4.54%)
Breast Cancer	3 (6.81%)
*Previous operations*	9 (20.45%)
*Side of breast reduction*	
Bilateral	41 (93.81%)
Unilateral	1 (2.27%)
Non-reported	2 (4.54%)
*Design of pedicle*	
Inferior pedicle	13 (29.54%)
Superomedial pedicle	3 (6.81%)
Superior pedicle	3 (6.81%)
Non-reported	27 (61.36%)
*Days to initial presentation of Pyoderma Gangrenosum*	6.5 days (2 days, 7 years)
*Days to diagnosis of Pyoderma Gangrenosum*	15.5 days (2 days, 7 years)
*Initial presentations of Pyoderma Gangrenosum*	
Fever	16 (36.36%)
Erythema	33 (75%)
Severe Pain	33 (75%)
*Surgical debridement*	30 (68.18%)
*Initial treatment of Pyoderma Gangrenosum*	
Antibiotics	34 (77.27%)
Hyperbaric oxygen therapy	1 (2.27%)
VAC Therapy	1 (2.27%)
*Culture results*	
Negative	25 (56.81%)
Corynebacterium striatum	2 (4.54%)
Staphylococcus aureus	5 (11.36%)
Pseudomonas aeruginosa	1 (2.27%)
Candida	1 (2.27%)
Non-reported	14 (31.81%)
*Immunological markers*	
Antinuclear antibody	3 (6.81%)
Hypogammaglobulinemia	1 (2.27%)
Antiparietal cell antibodies	1 (2.27%)

*VAC* vacuum-assisted closure, *SD* standard deviation

### Outcomes of Pyoderma Gangrenosum After Reduction Mammoplasty

The wound was healed by secondary intention among 26 (59.06%) patients. Skin grafting was performed for six (13.63%) patients, while three (6.81%) patients received skin substitutes. Four cases (9.09%) received hyperbaric oxygen, and three (6.81%) were treated with local wound care and primary closure. There was one case of recurrence four years after diagnosis of PG. The majority of cases showed complete healing with distorted breasts and hypertrophic scarring (Table 2).

## Discussion

PG is a rare condition after reduction mammoplasty and may result in wound deterioration shortly after the procedure. The condition does not respond effectively to antimicrobial agents and is commonly misdiagnosed, leading to progressive wounds and significant breast disfigurements [6, 12, 60]. The present systematic review gathered the reported cases of PG after reduction mammoplasty. The condition was developed with different age categories and among patients with or without comorbidities. Importantly, more than 20% of patients developed PG despite being subjected to previous abdominal or breast surgeries. These findings highlighted that the risk of PG is difficult to determine preoperatively, and the condition should be considered after reduction mammoplasty in patients with ulcerative lesions sparing the NAC. The outcome of PG is based mainly on early diagnosis and timely management with immunosuppressive therapies. The majority of cases were primarily diagnosed with local wound infection or dehiscence, for which antimicrobial agents were used, and unsuccessful first-line therapies were employed. Furthermore, more than half of the reported cases were subjected to surgical debridement, which may negatively impact the outcomes of PG and sophisticate the needed reconstructive procedures. These findings highlighted that PG after reduction mammoplasty is challenging to diagnose and mostly treated inappropriately, subjecting the patients to unnecessary and even ineffective therapies. The majority of cases showed healing of the lesions after a long period, yet with distorted breasts, poor scars, and a need for more reconstructive procedures.

The initial misdiagnosis of PG after reduction mammoplasty is common. The condition is mostly diagnosed as wound infection or necrotizing fasciitis, and patients are treated with antimicrobial therapy and wound debridement. Besides the delay in diagnosis, surgical debridement potentially exacerbates the condition and disfigures the breasts with the involvement of the surrounding healthy breast tissues. This was consistent with Ehlr et al., 2018 who reported single debridement in 49% of patients with PG after breast surgeries, and unnecessary removal of breast implants in 61% of patients. Despite that, wounds tended to progress after surgical debridement and after removal of the implants [7]. Brucato et al., 2024 review reported the lack of data regarding the diagnosis and management of PG after reduction mammoplasty, in which the majority of cases were diagnosed late after subjecting to unnecessary and often harmful interventions [27]. The development of PG in response to mechanical injury or surgery to the breast skin is considered a hyperactivity response. Surgical interventions before controlling the condition may worsen the PG [9, 35]. This systematic review highlighted the lack of evidence regarding PG after reduction mammoplasty, including cases with the involvement of NAC. Tuffaha et al., 2014 reported that surgical debridement accelerates PG and induces pathergy in healthy surrounding tissues that were yet uninvolved [9].

Prompt consultation with a dermatologist is necessary to confirm the diagnosis of PG, particularly with ineffective antibiotic therapy. The treatment needed to be initiated promptly before the results of histopathology or bacteriology, which may be non-specific for PG. A high index of clinical suspicion is needed to diagnose PG after reduction mammoplasty. The involvement of the breast is sharply demarcated, sparing the NAC [9]. Immunosuppressive therapies should begin immediately once the diagnosis of PG is established. Timely initiation of immunosuppressive therapy leads to dramatic improvement in wound activity. All further surgical interventions should be commenced under the coverage of immunosuppressive therapies and after lesions become quiescent. Noteworthy, reconstruction of wounds associated with PG is mostly unnecessary, and complete wound healing tends to occur with medical treatment only. The majority of cases in our review showed complete healing with secondary intention, and a limited number of cases needed reconstruction after controlling the condition. Pop et al., 2024 recommended an individualized treatment plan for patients with PG after breast surgery, emphasizing the need for timely diagnosis, meticulous planning, and comprehensive care to optimize patient outcomes [61].

The commonest conditions associated with PG after reduction mammoplasty were inflammatory bowel disease and breast cancer. Nearly 9% of patients had either Ulcerative colitis or Crohn’s disease. This was consistent with Ehlr et al., 2018 who reported that 8% of patients with PG after breast surgery were associated with inflammatory bowel disease [7]. Paradoxically, Zuo et al., 2015 reported that nearly 50–80% of patients with PG have concomitant systemic disease, mostly inflammatory bowel disease, rheumatologic disease, or haematological disorders [6]. In the present systematic review, only five patients had positive immunological markers. Zuo et al., 2015 reported an association between haematological disorders, inflammatory bowel disease, rheumatoid arthritis, and post-surgical PG [6]. The present systematic review gathered all cases with PG after reduction mammoplasty. However, the evidence retrieved from this review should be cautiously interpreted. The evidence obtained from case reports is limited regarding the long-term outcomes of PG.

## Conclusions

PG after reduction mammoplasty is a devastating condition associated with poor cosmetic and functional outcomes. The condition is difficult to diagnose, and the majority of cases are potentially subjected to ineffective medical therapy and unnecessary surgical debridement that worsen the prognosis of PG. Patients with existing immunological disorders and patients with a history of breast cancer were at higher risk of developing PG after reduction mammoplasty. The risk of PG after reduction mammoplasty still existed despite undergoing previous breast or abdominal surgeries. Patients with post-reduction mammoplasty PG mostly presented with erythema, severe pain, and fever one week since surgery. PG should be considered after reduction mammoplasty in patients with ulcerative lesions sparing the NAC.

## Data Availability

The datasets used in the present study are available from the first author and corresponding authors on reasonable request.
